# Chemical Compositions and Sources Contribution of Atmospheric Particles at a Typical Steel Industrial Urban Site

**DOI:** 10.1038/s41598-020-64519-x

**Published:** 2020-05-06

**Authors:** Guiqin Zhang, Chun Ding, Xiaojing Jiang, Guang Pan, Xiaofeng Wei, Youmin Sun

**Affiliations:** 1 School of Municipal and Environmental Engineering, Shandong Jianzhu University, Jinan, China; 2 Atmospheric environment department, Shandong Provincial Eco-environment Monitoring Center, Jinan, China

**Keywords:** Environmental sciences, Environmental social sciences

## Abstract

Online monitoring concentrations of PM at five sites were obtained from 01/01/2016 to 31/12/2016 in Laiwu, China, and PM_2.5_ filters were manually sampled for total 34 days at the same sites in four seasons in 2016. PM pollution sources, including soil dust, urban dust, construction dust, coal-fired power plants dust, steel plant dust and motor vehicle exhaust dust were sampled, respectively. The overall mean PM_2.5_/PM_10_ ratio (0.57) in Laiwu was at a relatively lower level compared with that in other Chinese cities, which was higher in winter, indicating fine particulate was the main contributor of atmospheric pollution in this period. NH_4_^+^ mainly existed in the form of NH_4_NO_3_ and (NH_4_)_2_SO_4_ during the sampling periods. Higher sulfate and NH_4_^+^ concentrations were in summer while higher nitrate concentrations prevailed in winter. The annual value of OC/EC was (5.38 ± 1.70), higher in summer and lower in winter, and the calculated SOC/OC value (%) was (43.68 ± 12.98)%. The characteristic components were Si, Fe and Ca in urban dust and soil dust; Ca, Mg, and NH_4_^+^ in construction dust; Fe, Ca and SO_4_^2−^ in steel dust; OC, EC and Si in motor vehicle exhaust dust; SO_4_^2−^, Al and NH_4_^+^ in power plant dust. Compared with other cities at home and abroad, it was found that the concentrations of metal elements in Laiwu were significantly higher than those in foreign cities, and at a medium level in China. With the improved CRAESCMB model, the urban dust was regarded as the receptor and the source of PM_2.5_ and apportioned its secondary sources contributions to PM_2.5_. The CMB results showed the contributions of secondary sources including sulfate (17%), nitrate (17%) and SOC (13%) to PM_2.5_ accounted for nearly half of all sources. Therefore, more attentions should be paid on secondary sources from the primary emission sources of the motor vehicle exhaust, coal combustion sources especially.

## Introduction

In recent decades, the rapid development of China’s economy, which has involved extensive industrialization and urbanization, has triggered many pollution problems. In particular, there has been an increase in the number of haze or smog episodes driven by elevations in atmospheric particulate matter (PM), especially particles with aerodynamic diameters <2.5μm (PM_2.5_) and 10μm (PM_10_)^1,2^. It is commonly known that PM pollution is associated with poor atmospheric visibility, high health risks, and global climate change, light extinction, and traffic accidents at the regional as well as local scale^3–5^. To improve air quality, the Atmospheric Pollution Prevention and Control Action Plan was enacted by the Chinese government (Chinese Ministry of Environmental Protection) in 2014. Since then, air quality across the country has improved greatly. However, air pollution in many cities containing industrial locations remains severe. To assist with such efforts, many scholars have carried out studies of the characteristics of atmospheric pollution, which have provided a basis for air pollution control and have facilitated sources analyses^6–8^. Researchers also found that PM_2.5_ concentrations exhibited seasonal variations, wherein the levels were the highest in winter and lowest in summer^9^. Chemical compounds of PM are known to contain ionic species, carbonaceous species, and metals and metalloids^10^. Secondary inorganic species, such as NO_3_^−^, SO_4_^2−^, and NH_4_^+^, which are water-soluble ions, are also known to be present in PM_2.5_ and can comprise a large fraction of PM in the atmosphere^11,12^. Organic carbon (OC), composed of thousands of organic compounds, originates from both natural and artificial sources. Element carbon (EC), with relatively stable chemical properties, is directly emitted from primary combustion and influences the global climate system through its impact on radiative forcing. High correlations between OC and EC have been found in PM_10_ and PM_2.5_^13^. It is generally acknowledged that local emissions remain an important factor affecting environmental quality, EPA CMB (Chemical Mass Balance) receptor model is a sources resolution technique to indicate the contribution of various pollution sources to receptors^14,15^, and has been extensively used for the apportionment of PM in the United States, Europe and Asia^16–20^. Although many researchers have conducted pollution characterization and sources assessment studies of PM_10_ and PM_2.5_ in ambient air, overall, these works were mainly conducted in developed cities and fast-developing areas^21–23^. Presently, there have been few detailed studies on atmospheric PM pollution in less urbanized and slow-developing cities^6^. Such studies are necessary because regional atmospheric environments are interconnected, and the ambient air in one city can affect the air quality in other cities via meteorological transport processes. In order to better evaluate the present situation of ambient air in a less developed city, a comprehensive study of the air pollution characteristics and sources of Laiwu must also be conducted.

Laiwu, an urban residential area of a typical steel industrial city in China, are investigated in this study. To the best of the author’s knowledge, few literatures has reported pollutant results for Laiwu. Laiwu, a medium-sized urban area, is located in the center of Shandong Province at the eastern foot of Mount Tai. This area is an important steel and energy production base in northern China and has experienced rapid development in recent years. Presently, it is one of the most heavily polluted areas in Shandong. Laiwu covers an area of 2,422 km^2^ and has a population of more than 1.27 million. Coal is the main energy source, and industry consumption accounts for more than 75% of the coal use. Many steel industries, located in the southern, western and northern parts of the city, have different producing processes, such as steel making, iron making, sintering and coking. Two major coal-fired power plants are located in the northeast and central of the city.

In this paper, the characteristics of atmospheric pollution and the chemical compositions of the particles during the monitoring period of one year in Laiwu were analyzed comprehensively. Hourly online monitoring data for PM_2.5_ and PM_10_ at different sites in Laiwu were obtained from the air quality monitoring network for the analysis, and manually sampled monitoring data were also collected. These data enabled us to gain a better understanding of the characteristics and chemical compositions of air pollutants in Laiwu. In addition, the composition spectrum of environmental receptors and seven sources of pollution in Laiwu City were also investigated, including water-soluble ions, inorganic elements and carbon components. The improved Chemical Mass Balance (CMB) model was used to analyze the sources distribution and air pollution levels in Laiwu were compared to those of other major cities. The results were helpful for the government to take effective PM pollution control strategies.

## Materials and Methods

### Data and samples collection

**Figure 1:**
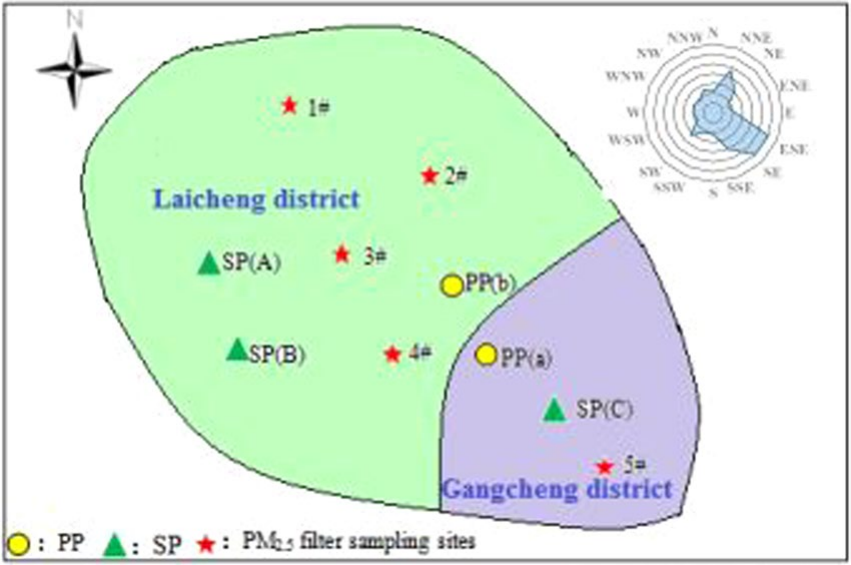
Sketch map of sampling sites chosen in this study (SP and PP stands for steel plant and power plant, respectively).

According to the relevant requirements of the Technical Specifications for Environmental Air Quality Monitoring Points (Trial) (HJ664-2013), fully considering complex factors such as climate, geographical conditions and pollution sources of Laiwu City, five sampling sites (1# Old apartment, 2# New first school, 3# Technical college, 4# Vegetable oil plants, and 5# Steel Environmental Protection Agency) were selected for obtaining online PM_2.5_ and PM_10_ and sampling manually the filters for PM_2.5_ chemical compositions, located in the different direction of the city, affected by the steel industries and power plants. Figure 1 shows the location of Laiwu and the distribution of ambient air sampling sites. Total 170 valid quartz filters (pall 7203, φ90mm) and 170 valid organic PTFE filters (Whatman, φ90mm) of PM_2.5_ receptor samples were respectively obtained. These PM_2.5_ filter samples were collected for 34 days at every site in 2016 (January 18–24, May 6–17, August 9–15, and October 29–November 5), representing collection in winter, spring, summer, and autumn. Each PM_2.5_ filter sample was collected for 24 hours using a median-flow particle samplers (Tianhong, Wuhan, Co. Ltd) with flow rate of 100 L/min. Model 602 Beta Plus dual-channel particulate matter automatic monitors (API Corporation, USA) were used as the online monitoring instruments for PM_10_ and PM_2.5_. The online hourly monitored concentrations of PM_10_ and PM_2.5_ at the same five sites as manual samples in Laiwu were obtained from 01/01/2016 to 31/12/2016, which indicated the PM pollution temporal characteristics and levels in a typical steel urban site for the whole year.

According to the Technical method guide for analytical monitoring of ambient air particle sources of China^24^, fully considering local air pollutant emission inventory, the emission pollution sources of PM_2.5_, including soil dust, urban dust, construction dust, power plants dust, steel dust and motor vehicle exhaust, were collected respectively. Total 86 valid quartz filters and 86 valid Telfon filters of all the emission sources samples respectively were obtained. Total 6 soil dust samples were collected by shovels in upwind and downwind main directions, 2 construction dust samples and 8 urban dust samples at every season were collected by vacuum cleaners, and pretreated by 140 mesh sieves, finally 32 quartz filters and 32 Telfon filters (φ47mm) were obtained by a resuspended chamber designed by Institute of Atmospheric Physics, Chinese Academy of Sciences, there are 8 pieces PM_2.5_ particle nozzles with the flow rate of 5 L/min, and only 1 gram sample is needed). Moreover, there were three kinds of emission sources collected directly by a diluting channel sampling equipment with the flow rate of 16.67 L/min made by Qingdao Laoshan Ltd, China) to obtain quartz filters and Telfon filters (φ47mm), including 12 quartz filters and 12 Telfon filters samples from different generator sets power plants, 24 quartz filters and 24 Telfon filters samples from sintering, coking and steelmaking steel plants and 18 quartz filters and 18 Telfon filters samples from gasoline and diesel motorvehicle.

### Samples pretreatment and chemical composition analyse

One quarter of a quartz filter sample was used to analyze the ions (NH_4_^+^, NO_3_^−^, SO_4_^2−^, F^−^ and Cl^−^) and one quarter to analyze OC/EC, the rests as other purposes. The selected filters were cut by ceramic scissors for four parts equally and then one quarter was soaked in 50 mL colorimetric tube with 20 mL de-ionized water for 6 h, extracted for 60 min in an ultrasonic cleaner, and filtered through a syringe filter (pore size 0.45 µm) to remove the insoluble materials and then was transferred and fixed to 50 mL in the volumetric flask for the determination of ammonium and anions. An area of 0.526 cm^2^ punched by one quarter of each quartz filter sample was used to analyze carbon composition (OC and EC). The OC and EC fractions were produced in a helium atmosphere at 140–580 °C and 580–840 °C, respectively. Each day, the instrument was baked for 30 min to remove residual carbon material before sample analysis. A CH_4_/CO_2_ standard gas was used for the calibration of the instrument before and after sample analysis.

Organic filters were used for the determination of elements. One half of one filter sample was put into the nickel crucible and ashed completely at 550 °C for 2 h in the muffle furnace and then melted at 500 °C in alkali fusion process (sodium hydroxide) for 10 minutes in the muffle furnace, finally, the volume was fixed with a certain proportion hydrochloric acid solution to analyze Si and the other part of one organic filter was placed into PTFE digestion vessel for acid treatment(2 ml hydrofluoric acid and 6 mL hydrogen nitrate), then treated by microwave digestion apparatus (CEM Mars 6, USA) for 2 h following the setup routine to analyze metal elements (HJ777-2015). Nineteen elements were determined, including metal elements (Na, Mg, Al, K, Ca, Ti, V, Cr, Mn, Fe, Co, Ni, Cu, Zn, As, Cd, Ba, Pb) and Si. Table 1 shows the analysis methods and details of equipment used in this study.

**Table 1:** Analysis methods and instruments used for the filter samples.

No.	contents	Analysis method	Instrument
1	PM_2.5_ mass	Weight method	Metler Toledo AX205
2	Anion analysis	Ion chromatography	Dionex ICS-1000
3	Carbon analysis	Thermal-optical carbon analysis	Multiwavelength Carbon Analyzer DRI Model 2015
4	NH_4_^+^	Ultraviolet and visible spectrophotometry	Ultraviolet and Visible Spectrophotometer TU-1810
5	Metal elements	Inductively coupled plasma spectroscopy	ICP- MS (ICP-5000)
6	Si	Inductively coupled plasma spectroscopy	ICP- OES (EXPEC-7000)

### CMB Model

Based on the theoretical basis of the standard EPACMB 8.0 model, Chinese Academy of Environmental Sciences proposed a improved CRAESCMB model^25^, which assumes that urban dust source is regarded as both a receptor and an emission source by exhaustive fitting calculation method. The method is proved to require the ideal results^26^. Sources contribution rate to PM can be calculated as Eq. (1) and Eq. (2).

1 $$C=\mathop{\sum }\limits_{i=1}^{m}\mathop{\sum }\limits_{j=1}^{n}{F}_{ij}\times {S}_{j}$$

2 $${\eta }_{j}={S}_{j}/C\times 100 \% $$

In this equation, C is the concentration of the all chemical components in the atmospheric particulate matter of the receptor, μg/m^3^; F_ij_ is a measured value of the chemical component i in the particulate matter of the j source, g/g; S_j_ is the calculated concentration of the contribution of the j source, μg/m^3^; j is the number of the source, j = 1, 2… n; i is the number of chemical components, i = 1, 2… m; η_j_ is the contributing rate of each source.

### Quality control

Each PM_2.5_ filter was weighed three times with the 1/100000 analytical balance to ensure that the error was less than 0.05 mg each time. Comparing the PM_2.5_ mass concentration measured by the filter collected from the manual sampling instrument at the same sampling online sites and the same period, it was found that the measure deviation was within 10%, which ensured the filter samplings’ mass concentration accuracy. The accuracy of a standard curve is the key to experimental quality control. In this study, the component contents of the prepared mixed standard solution were measured at different five concentrations to draw standard curves; the correlation coefficient (R^2^) between five concentration levels of every ion, every element and carbon component was above 0.99997. The recovery rates of blank filter and samples added standard solution respectively by each ion were 90–110%, 82–108% and by each element were 89–113% and 85–116%, by OC/EC with glucose standard solution were all 80–115%, which meet the EPA requirements. Every sample was detected parallel for at least three times, which was used to calculate the uncertainty of every component with the relative standard deviation for the reproducibility test, which is no more than 10% deviations for ion and OC/EC, 5–10% deviations for metal elements and 10% for Si. At least 7 pieces Quartz and organic blank filters respectively were treated by the same sample treatment process and then used to quantify the limit of method detection (LOD) with 3.14 times the relative standard deviation. The LOD for the analyzed ions are between 0.01 μg/m^3^ and 0.085 μg/m^3^, for OC and EC respectively 0.1 μg/m^3^, for 19 elements between 0.009 and 0.270 μg/m^3^, for Si 0.010 μg/m^3^. For the detecting accuracy of ions, elements and carbon components, a blank filter was conducted analysis every 10 samples to control the quality.

The input data of the model consisted of the chemical composition spectra and its uncertainty of environmental receptors and emission sources. Its uncertainty is the standard deviation(SD) calculated by the all data of the same component. The results of CMB model are mainly evaluated by sum of squares of residuals (χ^2^), regression coefficient (R^2^) and mass percent (% mass). When χ^2^ <1, R^2^å 0.8 and 80% <mass percent <120%, the results of CMB analysis are considered to be better^27^. All of the calculation fit the model requirements.

## Results and Discussion

### Pollution characteristics of atmospheric particulate matter

Coefficient of Divergence (CD) of PM_2.5_ between each two sampling sites

In order to analyze the spatial difference of PM_2.5_ between each two sampling sites, the Coefficient of Divergence (CD) of PM_2.5_ were calculated seen as Table 2. CD was used to test the extent of spatial difference^28–30^. The CD was defined as follows:

3 $$C{D}_{jk}=\sqrt{\frac{1}{p}\mathop{\sum }\limits_{i=1}^{p}{\left(\frac{{x}_{ij}-{x}_{ik}}{{x}_{ij}+{x}_{ik}}\right)}^{2}}$$

where j and k represented two sampling sites, and p was the number of chemical components. x_ij_ and x_ik_ were the average mass concentration for a chemical component i at site j and k. Referring to the literature^28^, if CD < 0.6, the difference between different sites of a certain pollutant is greater; if 0.3 < CD < 0.6, the difference is general; if CD < 0. 3, indicating that it has a certain similarity. Our calculated CD was between 0.007 to 0.148, below 0.3, indicating that there were no obvious differences for PM_2.5_ at the five sampling sites. So, the average concentrations of PM at five sampling sites can be used to analyze the regional pollution characteristics of the fine particles in Laiwu, China.

**Table 2:** Coefficient of Divergence (CD) of PM_2.5_ between each two sampling sites.

	1#	2#	3#	4#	5#
1#	—	—	—	—	—
2#	0.148	—	—	—	—
3#	0.138	0.100	—	—	—
4#	0.115	0.146	0.098	—	—
5#	0.039	0.002	0.007	0.012	—

### Pollution characteristics of atmospheric particulate matter

**Figure 2:**
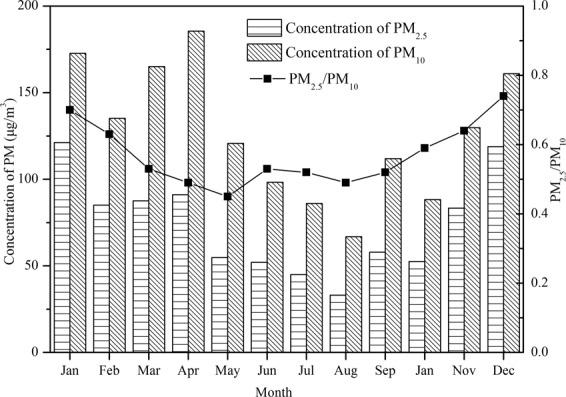
Monthly average concentrations of PM and the PM_2.5_/PM_10_ ratio.

The daily concentrations of PM_2.5_ and PM_10_ were averaged according to the hourly data from the five online monitoring sites. The monthly averaged values were computed by averaging the daily data in that month. The mean PM concentrations in Laiwu were averaged according to the data from all monitoring sites. The monthly average concentration ranges of PM_2.5_ and PM_10_ were 33.0–121.2 μg/m^3^ and 66.8–185.5 μg/m^3^, respectively, and the detailed results are illustrated in Fig. 2. The respective overall mean mass concentrations for PM_2.5_ and PM_10_ were 73.5 μg/m^3^ and 126.8 μg/m^3^, and the standard deviations were 28.6 μg/m^3^ and 38.2 μg/m^3^. The lowest concentrations of PM_2.5_ and PM_10_ were both observed in August, while the highest concentrations of PM_2.5_ and PM_10_ were observed in January and April, respectively. Throughout the whole year, the PM_10_ concentrations were relatively high from March to May during the spring period, and the PM_2.5_ concentrations were relatively high in January and December. These concentrations were mainly attributed to the climatic conditions and local emissions of Laiwu City. In spring, it often occurred the pollution of coarse particles such as road dust, soil dust, and construction dust because of the sandstorm caused by the higher wind speed^31^. In the cold and dry winter, the large amount of coal combustion by residents’ heating, and the geographical conditions that surrounded by mountains on three sides of the city, also tended to cause the accumulation of fine particles^32^. Compared to other cities in China (Table 3), the annual concentration of PM_2.5_ in Laiwu was at the same level as that of Tangshan, higher than that of Beijing (55 μg/m^3^), Shijiazhuang (65.1 μg/m^3^) and Zhuhai (34.4 μg/m^3^). The annual concentration of PM_10_ in Laiwu was lower than most neighboring province cities except Tianjin (86.6 μg/m^3^). In comparison with cities in same Province, its PM_2.5_ level was lower than Heze (109.1 μg/m^3^), and higher than Yantai (64.1 μg/m^3^). According to the Ambient Air Quality Standard (GB 3095–2012), except August, the PM_2.5_ and PM_10_ concentrations both exceeded their standard second grade limits (35 μg/m^3^ and 75 μg/m^3^, respectively). Hence, as a small city in China, its PM_2.5_ pollution was necessary to focus on.

**Table 3:** Comparison of air pollution levels between Laiwu and other cities.

Urban site	Sampling time	PM2.5 (μg/m^3^)	PM10 (μg/m^3^)	References
Laiwu	2016.01–2016.12	73.5 ± 28.6	126.8 ± 38.2	This article
Beijing	2016.01–2016.12	55	—	^67^
Beijing	2015.09–2016.08	—	144.75	^68^
Tianjin	2010.01–2010.12	—	86.6	^69^
Zhengzhou	2014.10–2015.07	146	214	^70^
Tangshan	2016.01–2016.12	74.1	—	^67^
Shijiazhuang	2016.01–2016.12	65.1	—	^67^
Shijiazhuang	2013.01–2013.12	—	303	^71^
Jinan	2010.01–2010.12	147.6	—	^72^
Qingdao	2007.08–2008.05	86.6	120	^73^
Heze	2015.08–2016.04	109.1	—	^74^
Yantai	2016.12–2017.10	64.1	—	^75^
Standard	—	35	75	(GB 3095–2012)

The variation in the PM_2.5_/PM_10_ ratio ranged from 0.45–0.74, and the overall mean value was 0.57. Zhang *et al*. investigated the PM_2.5_/PM_10_ in typical urban areas of Beijing-Tianjin-Hebei, Yangte River Delta and Pearl River Delta in 2016, it was found that the PM_2.5_/PM_10_ was ranged from 0.585 to 0.841, and above 0.70 of cities in Beijing-Tianjin-Hebei region^33,34^. Compared with these cities, PM_2.5_/PM_10_ ratio in Laiwu was at a relatively lower level. The overall temporal trend of the pollutant ratios showed a “U”-type distribution, wherein higher PM_2.5_/PM_10_ ratios were detected in December, January, and February and lower PM_2.5_/PM_10_ ratios were detected in March and May. These data suggest that fine particulates play an important role in the air pollution in winter. So it can be concluded that PM_2.5_ was the main pollutant in winter, contributing to the decline in visibility and heavy pollution levels in Laiwu.

### PM_2.5_ chemical compositions

#### Estimation of the existence forms of NH_4_^+^

In order to better explore the relationship between PM and air pollution, the existence of different forms of NH_4_^+^ in PM was investigated. The ratios of the measured NH_4_^+^ and calculated NH_4_^+^ were obtained, and the specific existence of NH_4_^+^ in NH_4_NO_3_, NH_4_HSO_4_, and (NH_4_)_2_SO_4_ was determined. A Three-phase diagram was used to show the existence of NH_4_^+^ ^35^. If SO_4_^2−^, NH_4_^+^, and NO_3_^−^ exist in the forms of NH_4_NO_3_ and NH_4_HSO_4_, the estimated concentration of NH_4_^+^ can be calculated according to Eq. (4). If SO_4_^2−^, NH_4_^+^, and NO_3_^−^ exist in the forms of NH_4_NO_3_ and (NH_4_)_2_SO_4_, the estimated concentration of NH_4_^+^ can be calculated according to Eq. (5). The calculation formulas for NH_4_^+^ are as follows^36^:

4 $$[N{{H}_{4}}^{+}]=0.29[N{{O}_{3}}^{-}]+0.19[S{{O}_{4}}^{2-}]$$

5 $$[N{{H}_{4}}^{+}]=0.29[N{{O}_{3}}^{-}]+0.38[S{{O}_{4}}^{2-}]$$

**Figure 3:**
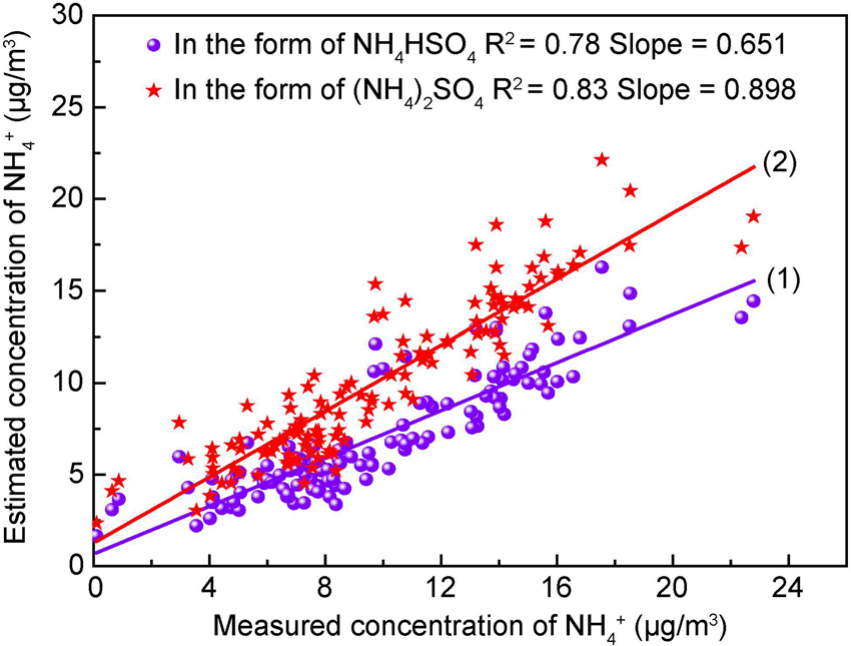
Existence of different forms of NH_4_^+^.

As shown in Fig. 3, when NH_4_^+^ was in the form of NH_4_HSO_4_, the slope of the fitted straight line was 0.651, and the square of the Pearson correlation coefficient (R^2^) was 0.78 (p value < 0.01, two-tailed test). When NH_4_^+^ was in the form of (NH_4_)_2_SO_4_, the slope of the straight line was 0.898, and R^2^ was 0.83 (p value < 0.01, two-tailed test). The slope in the form of (NH_4_)_2_SO_4_ was closer to 1 than NH_4_HSO_4_, hence NH_4_^+^ existed mainly in the form of NH_4_NO_3_ and (NH_4_)_2_SO_4_ during the four sampling periods.

#### Proportions of water-soluble secondary ions

**Figure 4:**
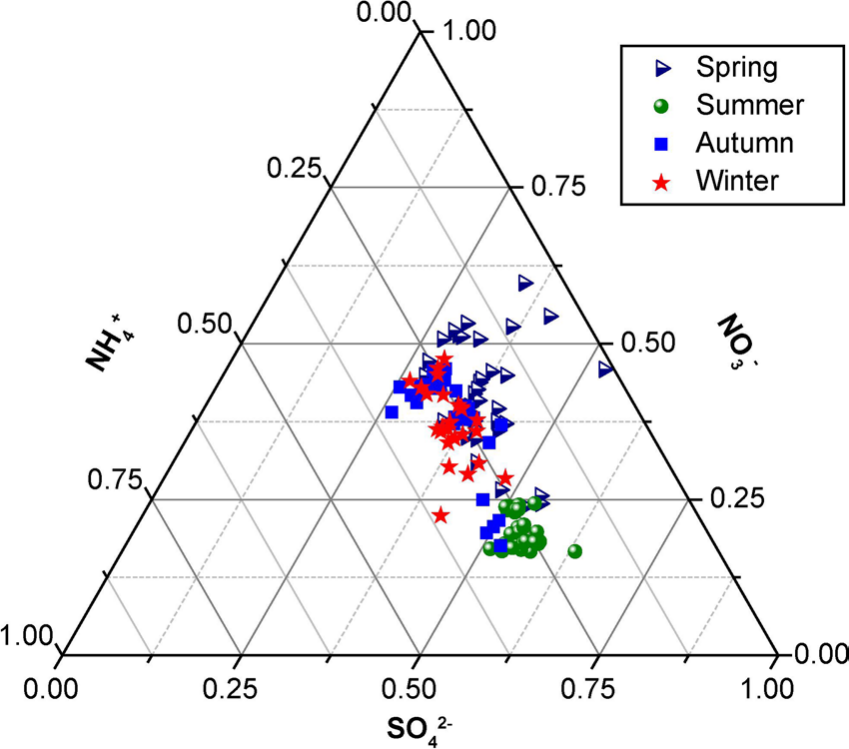
Proportions of SO_4_^2−^–NO_3_^−^–NH_4_^+^ during different seasons.

Using the weighted relative content data from the five manual sampling sites, the proportions of water-soluble secondary ions SO_4_^2−^, NH_4_^+^, and NO_3_^−^ were analyzed for the four different seasons. Proportions of the secondary ions during different seasons were characterized by a ternary phase diagram, and the detailed results were shown in Fig. 4.

As shown in Fig. 4, the data for the secondary water-soluble ions were mainly concentrated in the middle of the graph for all four seasons. During spring, NO_3_^−^ was the main component, with values exceeding 50%. The proportion of SO_4_^2−^ mainly ranged between 25% and 50%, and the NH_4_^+^ content was the lowest (less than 25%). The SO_4_^2−^ content in summer exceeded 50%, while that of NH_4_^+^ ranged between 18% and 37%. The NO_3_^−^ content was the lowest, with values below 25%. Therefore, the main ions present were SO_4_^2−^ and NH_4_^+^, and (NH_4_)_2_SO_4_ was the main form. Lesser amounts of NH_4_NO_3_ were also present in spring and summer. The SO_4_^2−^ content mainly ranged from 25% to 55% in autumn, with only a few values exceeding 50%. The NO_3_^−^ content ranged between 15% and 50%, and that of NH_4_^+^ was below 37%. The ratios of SO_4_^2−^ and NO_3_^−^ were roughly the same in winter, with values ranging from 25% to 50%. The NH_4_^+^ content ranged from 24% to 35%. Therefore, the ions existed mainly in two forms, namely, (NH_4_)_2_SO_4_ and NH_4_NO_3_, in autumn and winter. These results are consistent with those from earlier research^37^, which was conducted in autumn over southern Hebei, China. According to a study at an urban site in Karachi, Pakistan, 4.4% (NH_4_)_2_SO_4_ existed in PM_2.5_^38^.

#### OC/EC and SOC

OC/EC can be used not only to assess the degree of secondary pollution, but also to speculate on the sources of carbon components. OC/EC indicates vehicle exhaust in the range of 1.0–4.2, and fired sources in the range 2.5–10.5^39,40^. When it exceeds 2.0, it is suggested there is SOC (Secondary Organic Carbon) generated by the secondary reaction^41,42^. The annual value of OC/EC was calculated to be 5.38 ± 1.70, higher than 4.2 and lower than 10.5, indicating the sources of carbon components was fired sources such as power plants and steel industries. The seasonal average value of the ratio was (4.54 ± 2.07), (6.07 ± 1.13), (5.01 ± 1.28) and (5.87 ± 1.86), respectively. The higher OC/EC in summer may be attributed to high temperatures, strong solar radiation and the easy reaction of hydrocarbons in the atmosphere, which significantly promotes the formation of SOC^43^. The SOC can be calculated by the Eq. (6) as reported in the literature^42,43^. The annual value of was calculated to be (8.22 ± 3.93) μg/m^3^ for SOC, and (43.68 ± 12.98)% for SOC/OC, which was higher in summer and lower in Spring, which was in consistent with the OC/EC.

6 $$SOC=OC-{(OC/EC)}_{min}$$

#### Chemical composition spectrum of PM_2.5_ and its pollution sources

**Figure 5:**
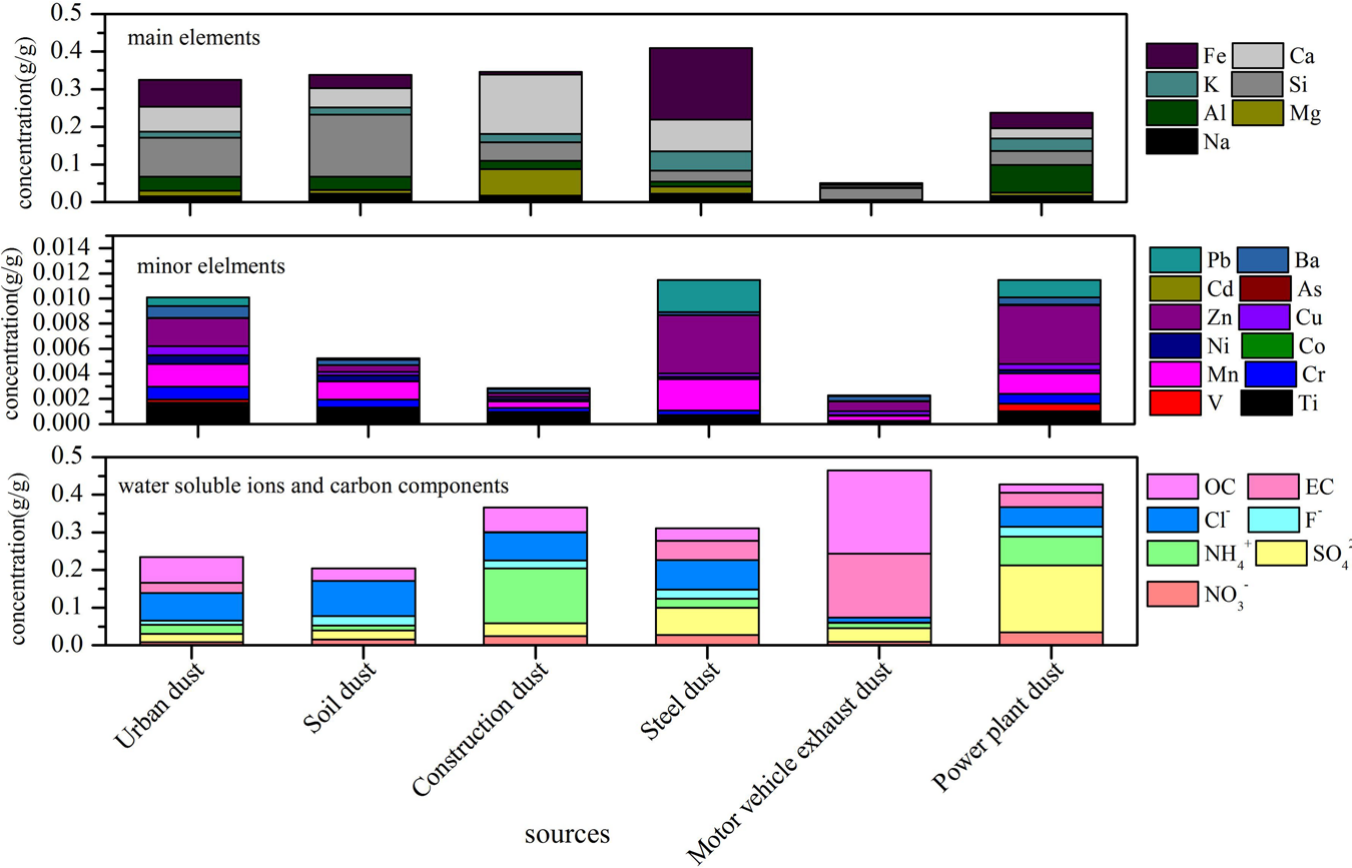
Composition spectrum of sources.

**Figure 6:**
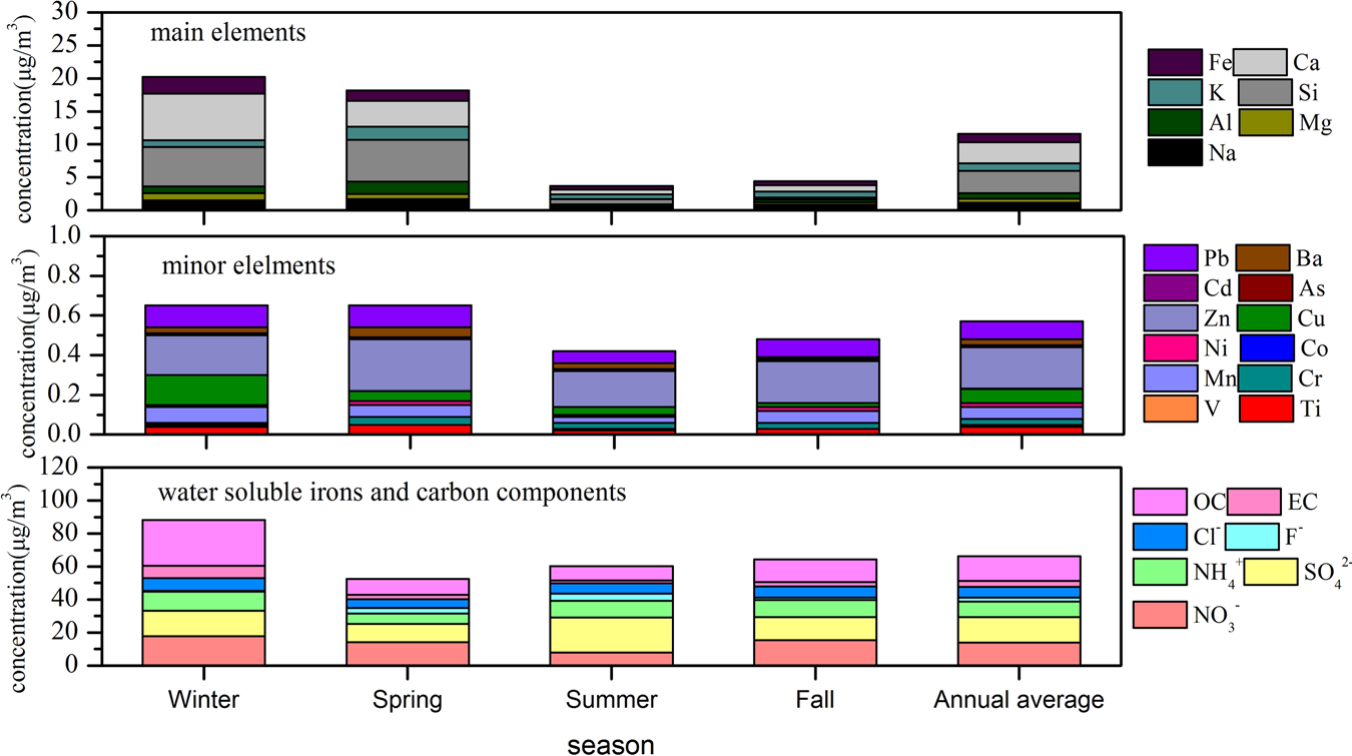
Composition spectrum of PM_2.5_.

Every chemical composition at five sites in different seasons for PM_2.5_ filter samples was averaged as the environmental receptor. Road dust, urban dust, soil dust, construction dust, steel dust, power plants dust and motor vehicle exhaust dust were regarded as pollution sources. Compositional spectrum of environmental receptors and sources were established respectively, as shown in Fig. 5 and Fig. 6.

#### Composition spectrum of sources

As shown in Fig. 5, for main elements, the concentration of Si in urban dust and soil dust was higher (0.10 g/g and 0.16 g/g) and the concentration of Ca, Fe were also higher, which showed the similar and complex sources. Lai *et al*. reported the same results^44^. However, Si is an element associated to crustal dust and coal-fired power plants^45^. For construction dust, steel dust, power plant dust, the higher main element was Ca, Fe and Al, respectively, which in consistent with the results of other literatures^46^, while Al and Ca could be also associated to road dust and soil dust^45^. For minor elements, the concentration of Zn and Mn was high in most sources especially steel dust and power plant dust, showing the complexity of their sources, in consistent with the conclusion of relevant literatures^46^. Ti was abundant in natural sources including urban dust, soil dust and construction dust, and literatures reported the similar results^47^. Pb was rich in steel dust and power plant dust, and former researches showed that, with the widespread use of unleaded gasoline, the contribution of automobile exhaust to Pb grew to decline, while industrial emissions became the main contributor^48^. The concentration of Cr was high in urban dust and power plant dust, references also showed the industrial productions were the crucial emission sources to Cr^48^. For water soluble ions, the concentration of NH_4_^+^ (0.15 g/g) in construction dust was higher, which showed that construction dust was also affected by secondary transformation. For power plants dust, SO_4_^2−^ was the highest concentration component (0.18 g/g), in consistent with the conclusion of the reported literature^48^. The higher components of motor vehicle exhaust dust were OC and EC, seen as the main chemical component. The characteristic components were Si, Fe and Ca in urban dust and soil dust; Ca, Mg and NH_4_^+^ in construction dust; Fe, Ca and SO_4_^2−^ in steel dust; OC, EC and Si in motor vehicle exhaust dust; SO_4_^2−^, Al and NH_4_^+^ in power plant dust.

#### Composition spectrum of PM_2.5_

As seen in Fig. 6, for main elements, the concentrations of Si, Ca, Fe, Al were higher than that of other elements, with annual average values of 3.35, 3.15, 1.32 and 0.87 μg/m^3^, respectively, especially in winter and spring in accordance with the main components of soil dust, construction dust, steel dust and power plants dust in Fig. 5. The concentration of other main elements ranged between 0.60 and 1.17 μg/m^3^. For minor elements, the annual average concentration of Zn (0.210 μg/m^3^) was much higher *compared with the value of* heavy metals elements such as Pb, V, Mn, Ni, Cd between 0.003 and 0.093 μg/m^3^. As shown in Fig. 5 the concentration of these minor elements all appeared higher in power plants dust and steel dust. The results were consistent with literature reports that these elements had high content in power plants dust, steel dust, or motor vehicles exhaust^49–51^. The annual average concentration of heavy metals elements with high toxicity was all below the WHO annual concentration limits^52^. This showed that heavy metals pollution in Laiwu was not serious. Compared with other cities at home and abroad, it was found that the concentrations of main elements in Laiwu were significantly higher than these in foreign cities^53–55^, and at a medium level in China, higher than that in Hangzhou^56^, Yantai^57^ and Haikou^58^, while lower than that in Ningbo^59^, Tianjin^60^ and Qingdao^61^, while the concentration levels of minor elements in Laiwu were much lower than these cities^53–61^, indicating a slightly pollution status of minor elements. The seasonal distributions of both main elements and minor elements were characterized by higher concentration in winter and spring. This may be mainly due to the large amount of pollutants discharged by the increase of coal combustion in winter, and the impact of dust weather in spring, which is consistent with the results of relevant references^62,63^. For water soluble ions, the annual concentrations of SO_4_^2−^, NO_3_^−^ and NH_4_^+^, with the value of 15.42, 13.84 and 9.53 μg/m^3^, respectively, were higher compared with the value of F^−^ and Cl^−^ between 2.47 and 6.48 μg/m^3^. SO_4_^2−^, NO_3_^−^ and NH_4_^+^ were the secondary pollutants and mainly concentrated in power plants dust, motor vehicle exhaust and construction dust compared to Fig. 5. OC and EC also showed a relatively high annual concentration, 14.91 and 3.27 μg/m^3^, respectively. All water soluble irons and carbon components showed the higher concentration in winter except for SO_4_^2−^ that higher in summer, mainly due to the fact that the higher temperature in summer was beneficial to the transformation of SO_4_^2–64^.

#### CMB Sources apportionment results

**Figure 7:**
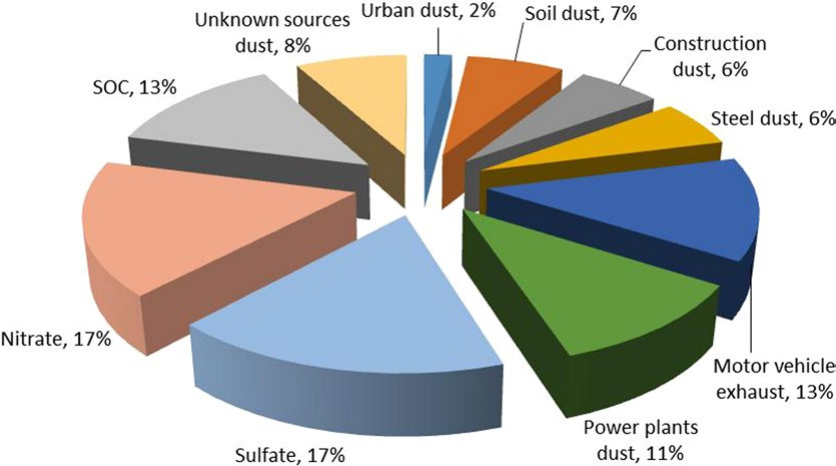
Sources contribution apportionment results for PM_2.5_ combined CMB model.

Based on the Eq. (3), CD of every chemical component between each two sampling sites for four seasons were calculated. The CD ranged from 0.36 to 0.50 in Si, from 0.11 to 0.57 in metal elements, from 0.09 to 0.36 in OC, from 0.15 to 0.48 in EC, from 0.12 to 0.38 in irons, all below 0.6. In general, the larger CD were observed in metal elements between S3 and S4, indicating the statistic significant differences likely due to the influence of human traffic and urban emission. The analysis of CD gave an reasonable agreement that the data can be averaged as an input dataset to the CMB model. SOC was regarded as an emission source input to CMB model to obtain the SOC source contribution in PM_2.5_. The results of sources apportionment were shown in Fig. 7. The contribution rates of the primary pollution sources apportionment for power plants dust and steel dust were 11% and 6%, respectively. Worthily, motor vehicle exhaust accounted for 13%, which should be paid more attention by the local government. The contribution rates of surface resuspension dust sources including soil dust, construction dust and urban dust were 7%, 6% and 2%, respectively, a total of 15%. Moreover, the annual contribution rate of secondary generation sources including sulfate, nitrate and SOC of PM_2.5_ was 17%, 17% and 13%, respectively, which were nearly half of the total sources, and the primary sources of these sources should be taken care in the future research study. Kim used the receptor Model (PMF model), and Contini assosciated PMF Model with CMB Model to analyze the sources of particulate matter^65,66^. They both found that secondary generation sources were the most important contributor to particulate matter. Therefore, more attention should be paid on secondary generation sources as well as the motor vehicle exhaust and road dust caused by their transportation.

## Conclusions

Online monitoring data for atmospheric particulate matter (PM) during the whole year in 2016 and manual filters sampling of PM_2.5_ during four seasons in Laiwu, Northern China, were used to study the characteristics and chemical compositions of pollutants in PM at a typical steel industrial urban site. PM pollution sources, including soil dust, urban dust, construction dust, coal-fired power plants dust, steel dust and motor vehicle exhaust dust were sampled, respectively. The results showed that the average concentrations of PM_10_ and PM_2.5_ were (126.8 ± 38.2) µg/m^3^ and (73.5 ± 28.6) µg/m^3^, respectively. The PM_2.5_/PM_10_ ratio was the highest in winter, indicating PM_2.5_ was the dominant pollutants in this period. Water-soluble secondary ions mainly existed in the form of NH_4_NO_3_ and (NH_4_)_2_SO_4_ during the sampling periods. The annual value of OC/EC was 5.38 ± 1.70, higher in summer and lower in winter, and the calculated SOC/OC value was (43.68 ± 12.98) %. The characteristic components were Si, Fe and Ca in urban dust and soil dust; Ca, Mg and NH_4_^+^ in construction dust; Fe, Ca and SO_4_^2−^ in steel dust; OC, EC and Si in motor vehicle exhaust dust; SO_4_^2−^, Al and NH_4_^+^ in power plant dust, respectively. The CMB model results showed that the contribution rates of the primary pollution sources apportionment for power plants dust and steel dust were 11% and 6%, and the annual contribution rate of secondary generation sources including sulfate, nitrate and SOC to PM_2.5_ was 17%, 17% and 13%, respectively.

## Data Availability

The data of the compounds are available from the authors. 2020 by the authors. Submitted for possible open access publication under the terms and conditions of the Creative Commons Attribution (CC BY) license (http://creativecommons.org/licenses/by/4.0/).
